# Secreted miR-210-3p as non-invasive biomarker in clear cell renal cell carcinoma

**DOI:** 10.18632/oncotarget.18449

**Published:** 2017-06-13

**Authors:** Vincenzo Petrozza, Antonio Luigi Pastore, Giovanni Palleschi, Claudia Tito, Natale Porta, Serena Ricci, Chiara Marigliano, Manuela Costantini, Giuseppe Simone, Angelina Di Carlo, Michele Gallucci, Antonio Carbone, Francesco Fazi

**Affiliations:** ^1^ Department of Medico-Surgical Sciences and Biotechnologies, Sapienza University of Rome, Pathology Unit ICOT, Latina, Italy; ^2^ Department of Medico Surgical Sciences and Biotechnologies, Sapienza University of Rome, Urology Unit ICOT, Latina, Italy; ^3^ Department of Anatomical, Histological, Forensic & Orthopaedic Sciences, Section of Histology & Medical Embryology, Sapienza University of Rome, Rome, Italy; ^4^ Department of Medico-Surgical Sciences and Biotechnologies, Sapienza University of Rome, Latina, Italy; ^5^ Department of Radiological Sciences, Oncology and Pathology, Azienda Policlinico Umberto I, Sapienza University of Rome, Rome, Italy; ^6^ Department of Biosciences, Biotechnologies and Biopharmaceutics, University of Bari, Bari, Italy; ^7^ Department of Urology, Regina Elena National Cancer Institute of Rome, Rome, Italy

**Keywords:** microRNAs, biomarkers, ccRCC, miR-210-3p, urine specimens

## Abstract

The most common subtype of renal cell carcinoma (RCC) is clear cell RCC (ccRCC). It accounts for 70-80% of all renal malignancies representing the third most common urological cancer after prostate and bladder cancer. The identification of non-invasive biomarkers for the diagnosis and responsiveness to therapy of ccRCC may represent a relevant step-forward in ccRCC management. The aim of this study is to evaluate whether specific miRNAs deregulated in ccRCC tissues present altered levels also in urine specimens. To this end we first assessed that miR-21-5p, miR-210-3p and miR-221-3p resulted upregulated in ccRCC fresh frozen tissues compared to matched normal counterparts. Next, we evidenced that miR-210-3p resulted significantly up-regulated in 38 urine specimens collected from two independent cohorts of ccRCC patients at the time of surgery compared to healthy donors samples. Of note, miR-210-3p levels resulted significantly reduced in follow-up samples. These results point to miR-210-3p as a potential non-invasive biomarker useful not only for diagnosis but also for the assessment of complete resection or response to treatment in ccRCC management.

## INTRODUCTION

Renal cell carcinoma (RCC) represents 2-3% of adult malignancies [1], with a peak of incidence occurring between 60 and 70 years and a 3:2 male:female ratio [2]. The clear cell RCC (ccRCC) is the most common subtype of RCC and represents the third most common urological cancer after prostate and bladder cancer. The incidence of the disease has been steadily rising in Europe to over 30,000 new cases per year and its mortality rate has reached 40% [3]. The early diagnosis and complete surgical excision are the main factors contributing to a definitive treatment of ccRCC. Surgery represents the most important therapeutic option for ccRCC management [4], but nephrectomy of the primary tumor is curative only if the tumor is still localized in the kidney. Thus, for patients with advanced and metastatic ccRCC, surgery is often palliative [5]. Targeted therapy has recently emerged in ccRCC management considering also that ccRCC is usually resistant to both chemotherapy and radiotherapy. Tyrosine kinases or mTOR pathways inhibitors are drugs often used in advanced RCC [6, 7]; however, resistance frequently occurs after these targeted therapies [8]. A key challenge for the improvement of ccRCC management could derive from a deeper molecular characterization of this neoplasm that could greatly improve the diagnosis, prognosis and treatment choice [9]. The comprehension of microRNAs (miRNAs) contribution to tumorigenesis and the identification of miRNAs as potential biomarkers for diagnosis and therapy response are becoming relevant for cancer management [10, 11]. To date, a number of studies identified miRNAs differentially expressed between normal and neoplastic tissues in ccRCC as well as miRNAs secreted into blood and urine, suggesting that these small molecules may serve as non-invasive diagnostic, prognostic and surveillance markers in urological carcinomas [12–17]. The urinary miR-210 is currently under investigation as a potential tool for ccRCC management [18].

Recently, by using a retrospective cohort of 20 formalin-fixed paraffin-embedded (FFPE) tissues, we evaluated the levels of specific miRNAs (miR-21-5p, miR-210-3p, miR185-5p and miR-221-3p) differentially expressed in ccRCC *vs* matched normal tissues. We evidenced miR-21-5p and miR-210-3p as the most significantly up-regulated in this patient cohort, highlighting these onco-miRNAs as possible relevant players involved in ccRCC tumorigenesis [19]. To further support the potential clinical usefulness of these miRNAs in ccRCC management we started a prospective study. By using two independent cohorts of patients we evidenced that, among the miRNAs previously identified as up-regulated in ccRCC tissues, only miR-210-3p resulted significantly up-regulated in urine specimens collected from 38 ccRCC patients at the time of surgery compared to healthy donors samples. Of note, miR-210-3p levels resulted significantly reduced in follow-up samples, highlighting this onco-miRNA as potential biomarker useful not only for diagnosis but also to assess complete resection or response to treatment in ccRCC management.

## RESULTS

**Figure 1:**
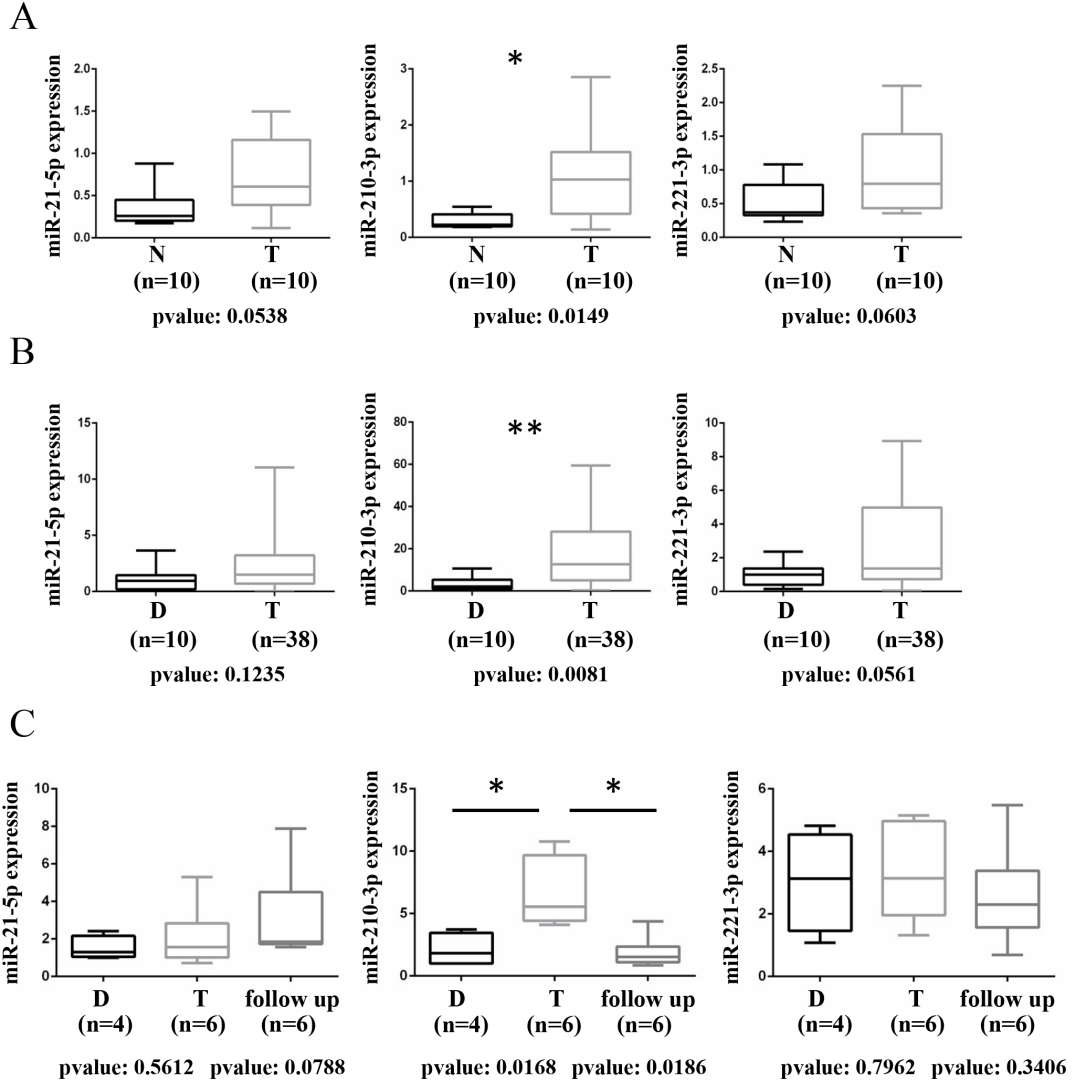
Evaluation of microRNAs levels in fresh frozen tissues and urine specimens from ccRCC patients **(A)** Box-plot showing the modulation of miR-21-5p, miR-210-3p and miR-221-3p in a cohort of 10 matched fresh frozen tissues from ccRCC patients. A total of 10 matched ccRCC tumor (T) and adjacent normal tissue (N) samples were analyzed by RT-qPCR. The expression value of each miRNA was normalized over the average of SNORD61, SNORD68 and RNU6-2 expression through z-scores method. p-value was calculated by paired t-test and a value of P≤0.05(*) was considered statistically significant. **(B)** Box plots showing the modulation miR-21-5p, miR-210-3p and miR-221-3p in urine specimens collected from 38 ccRCC patients at the time of surgery (Tumor) and from 10 healthy donors samples (Donors) by RT-qPCR and normalized relative to the expression level of Spike-In Control through ΔΔ CT method. p-value was calculated by unpaired t-test and a value of P≤0.01(**) was considered statistically significant. **(C)** Box plot showing the expression level of miR-21-5p, miR-210-3p, miR-221-3p in urine collected from 6 patients at time of surgery (T), during follow-up (15 months as median of time from surgery) and from 4 healthy donors samples by RT-qPCR and normalized relative to the expression level of Spike-In Control through ΔΔ CT method. p-value was calculated by unpaired t-test and a value of P≤0.05(*) was considered statistically significant.

By using a cohort of fresh frozen tissues obtained from 10 ccRCC patients undergoing surgical nephrectomy resection we evaluated the expression of the same miRNAs analyzed in our previous retrospective study [19]. The characteristics of ccRCC patients and tumor specimens are reported in the Methods section and summarized in Table 1. In agreement with the previous retrospective study we evidenced that miR-21-5p, miR-210-3p and miR-221-3p resulted up-regulated in these ccRCC *vs* matched adjacent normal fresh frozen tissues, while miR-185-5p and miR-145-5p did not show significant modulation (Figure 1A and Supplementary Figure 1A). Our data evidenced that miR-210-3p resulted the most significantly up-regulated miRNA in this patients cohort (p-value =0.0149) (Figure 1A).

**Table 1:** Clinical characteristics of patients with ccRCC

Gender/ age	Weight/height (kg/cm)	Smoking history	Serum creatinine (mg/dl)	Histology	Tumor grade (Fuhrman)	Pathologic stage	Tumor size (cm)	L. Nodes Inv.	Metast.	Surgery
M/52*	70kgx165cm	No	1.11	ccRCC	G2	pT2	8.0	Nx	Mx	Lap. Rad. Right Neph.
F/73**	65kgx160cm	No	0.88	ccRCC	G2	pT1a	2.0	Nx	Mx	Lap. Rad. Right Neph.
M/65*	75kg x170cm	No	1.00	ccRCC	G2	pT1a	2.0	Nx	Mx	Lap. Part. Right Neph.
M/74**	75kg x165cm	No	0.84	ccRCC	G2	pT1a	nd	Nx	Mx	Lap. Part. Left Neph.
M/84**	90kg x165cm	No	0.75	ccRCC	G2	pT1a	3.0	Nx	Mx	Lap. Rad. Right Neph.
M/70*	85kg x170cm	Yes	0.99	ccRCC	G2	pT3a	7.5	Nx	Mx	Lap. Rad. Left Neph.
M/70**	78kg x175cm	Yes	1.55	ccRCC	G2	pT1a	2.5	Nx	Mx	Lap. Part. Right Neph.
M/42**	60kg x170cm	Yes	1.13	ccRCC	G2	pT3a	9.5	N0	Mx	Lap. Rad. Left Neph.
F/36**	50kg x155cm	No	2.70	ccRCC	G2	pT1b	5.0	Nx	Mx	Lap. Rad. Left Neph.
M/81*	80kg x174cm	Yes	1.01	ccRCC	G2	pT1b	4.3	Nx	Mx	Lap. Rad. Left Neph.
F/64^†^	99kg x180cm	Yes	1.3	ccRCC	G3	pT3a	6	N0	Mx	Lap. Rad. Right Neph.
M/74^†^	73kg x160cm	Former	1.21	ccRCC	G3	pT1a	3	Nx	Mx	Lap. Part. Right Rob. Neph.
M/51^†^	85kg x185cm	Former	1.19	ccRCC	G3	pT2a	8	N0	Mx	Lap. Rad. Left Neph.
M/61^†^	92kg x165cm	Former	1.32	ccRCC	G3	pT3b	7	Nx	M1	Lap. Rad. Right Rob. Neph.
M/42^†^	n/a	Yes	1.36	ccRCC	G3	pT3a	7.5	Nx	Mx	Lap. Rad. Left Neph
M/74^†^	84kg x183cm	No	1.1	ccRCC	G3	pT3a	5.4	Nx	Mx	Lap. Part. Left Rob. Neph.
F/61^†^	n/a	n/a	1.06	ccRCC	G3	pT3a	5	Nx	M1	Lap. Rad. Right. Neph.
F/77^†^	78kg x165cm	No	1.1	ccRCC	G4	pT1b	6	Nx	Mx	Lap. Rad. Left Neph.
M/73^†^	90kg x175cm	No	1.4	ccRCC	G3	pT1a	4	Nx	Mx	Lap. Part. Left Rob. Neph.
M/62^†^	69kg x164cm	No	1.18	ccRCC	G2	pT2a	7.2	Nx	Mx	Lap. Part. Right Rob. Neph.
M/45^†^	69kg x170cm	Yes	1	ccRCC	G2	pT1a	2.5	Nx	Mx	Lap. Part. Right Rob. Neph.
F/62^†^	80kg x160cm	No	5.5	ccRCC	G2	pT1b	0.88	Nx	Mx	Lap. Part. Right Rob. Neph.
F/82^†^	65kg x155cm	No	0.66	ccRCC	G2	pT1a	3.5	Nx	Mx	Lap. Part. Right Rob. Neph.
M/53^†^	71kg x168cm	Yes	1.12	ccRCC	G4	pT1b	6	Nx	M1	Lap. Rad. Left Neph.
F/68^†^	80kg x160cm	Former	0.89	ccRCC	G2	pT1b	5	Nx	Mx	Lap. Part. Left Rob. Neph.
F/64^†^	n/a	n/a	1	ccRCC	G2	pT1a	3	Nx	Mx	Lap. Part. Left Rob. Neph.
F/78^†^	n/a	n/a	0.86	ccRCC	G4	pT3a	7.5	Nx	M1	Lap. Rad. Left Neph.
M/52^†^	60kg x169cm	Yes	0.79	ccRCC	G3	pT1a	3.5	Nx	Mx	Lap. Part. Right Rob. Neph.
M/53^†^	n/a	n/a	1.3	ccRCC	G4	pT3a	11	Nx	Mx	Lap. Rad. Right Neph.
M/83^†^	59kg x164cm	Yes	1.24	ccRCC	G3	pT3a	7.4	Nx	Mx	Lap. Rad. Left Neph.
M/84^†^	n/a	Yes	1.26	ccRCC	G4	pT2b	10.2	Nx	Mx	Lap. Rad. Left Neph.
F/48^†^	n/a	n/a	1.12	ccRCC	G3	pT1a	3.5	Nx	Mx	Lap. Part. Left Neph.
M/45^†^	n/a	n/a	1.16	ccRCC	G2	pT1b	4.2	Nx	Mx	Lap. Part. Left Rob. Neph.
M/72^†^	n/a	n/a	1	ccRCC	G3	pT1a	2.5	Nx	Mx	Lap. Part. Right Rob. Neph.
M/73^†^	n/a	n/a	1.08	ccRCC	G3	pT1a	2.6	Nx	Mx	Lap. Part. Right Rob. Neph.
F/49^†^	62kg x165cm	Former	1.2	ccRCC	G2	pT1a	3.5	Nx	Mx	Lap. Rad. Right Neph.
M/45^†^	80kg x175cm	No	1.26	ccRCC	G2	pT3a	11	N0	M1	Open Rad. Right Neph.
F/68^†^	70kg x171cm	Yes	1.7	ccRCC	G1	pT1b	5.5	Nx	Mx	Lap. Rad. Right Neph.

M: male; F: female; Metast: metastasis; Nx: regional lymph nodes cannot be assessed; N0: no regional lymph node metastasis; Mx: distant metastasis cannot be evaluated; M1: distant metastasis evaluated; Lap: laparoscopic; Rad: radical; Neph: nephrectomy; Part: partial; Rob: robotical; N/a: not available; *: Belonging to the first cohort; **: Belonging to the first cohort for which also follow-up samples are available; †: Belonging to the second cohort.

To investigate whether the miRNAs deregulated in ccRCC tissues may serve as useful clinical biomarkers, we selected miR-21-5p, miR-210-3p and miR-221-3p to evaluate their expression in 38 urine specimens collected at the time of surgery and during follow-up (with 15 months as median of time from surgery) from two independent cohorts of ccRCC patients; urine specimens from healthy donors were analyzed as well.

Of note, miR-210-3p resulted significantly up-regulated in urine specimens collected from ccRCC patients at the time of surgery, compared to healthy donors samples (Figure 1B), contrarily to miR-21-5p and miR-221-3p, which didn’t show increased levels in urine despite their trend of up-regulation observed in tumor tissues.

Importantly, miR-210-3p levels resulted significantly reduced during follow-up in six ccRCC patients of the same cohort, for which follow-up urine samples were available (Figure 1C). miR-21-5p and miR-221-3p resulted unchanged also in the follow-up samples (Figure 1C).

## DISCUSSION

The identification of molecular markers in body fluids (e.g., sera and urine), which can be used as non-invasive diagnostic, prognostic and surveillance markers in ccRCC management, is one of the most ambitious challenges in oncologic research [20]. Of note, also miRNAs are acquiring a great potential as novel cancer biomarkers in urological carcinomas [16, 21–25]. To date, the increased interest on non-invasive biomarkers, allowed by use of novel methodologies (such as next generation sequencing, single-cell sequencing approaches and digital PCR) has greatly improved the translational potential of miRNAs into clinical research [16]. However, the development of a standardized method considering different methodological drawbacks (including samples collection, processing and storage or RNA isolation, quantification and normalization) is strongly needed to support the strength of circulating miRNAs as useful biomarkers in ccRCC management [16, 25].

In this study we show that miR-210-3p that was previously evidenced as up-regulated in ccRCC *vs* matched normal tissues [19] also show increased levels in urine specimens from ccRCC patients when compared with healthy donors. Of note the urine expression levels of miR-21-5p and miR-221-3p resulted unchanged in these cohorts of ccRCC patients. These results not only support the identification of miR-210-3p as non-invasive biomarker for ccRCC management, as also suggested by Li and colleagues, but also highlight that only specific onco-miRNAs are accumulated in urine specimens of ccRCC patients. Of note, the urine level of miR-210-3p resulted significantly reduced during follow-up samples highlighting this onco-miRNA as potential biomarker useful not only for diagnosis but also to assess complete resection or response to treatment of ccRCC patients.

In conclusion, this study evidences a number of miRNAs, which are altered in ccRCC tissues and urine specimens, emerging as putative non-invasive biomarkers for ccRCC management. Further investigation including larger cohorts of patients will allow evaluating the associations existing between miRNAs levels and insurgence of metastasis, evidencing the strength of these biomarkers in the monitoring of tumor progression in ccRCC.

## MATERIALS AND METHODS

### Patients and urine samples collection

This study includes two independent cohorts of ccRCC patients (totally 38 patients) who underwent surgical resection between March 2015 and March 2017 (as described in Table 1). For the 10 ccRCC patients of the first cohort, fresh frozen-matched tumoral and normal peritumoral kidney tissues were also considered. Urine specimens were collected at the time of surgery from all the patients; for 6 of the ccRCC patients urine was collected also during follow-up (with 15 months as median of time from surgery). In particular, urine samples were frozen within 30 minutes from collection and stored at -80°C until RNA extraction. Patients included in the study were not treated with any neo-adjuvant therapy before surgery. The surgery procedures performed as curative treatment for these patients were: Laparoscopic Radical Right or Left Nephrectomy in 20 cases (52,63%), Laparoscopic Partial Right or Left Nephrectomy in 17 cases (44,73%) and Open Radical Right Nephrectomy in only 1 case (2,63%). Twenty-five patients were male (65.8%) and 13 patients were female (34.2%) with a median age of 64.5 years old (range 36-84) and a median Serum Creatinin concentration of 1.00 mg/dl (range 0.66-2.70). All the cases presented a clear cell histotype of RCC at the histological examination and according to Fuhrman’s grade classification, 19 cases (50%) were G2 grade, 13 cases (34.21%) were G3 grade, 5 cases (13.16%) were G4 grade and only 1 case (2.63%) was G1 grade. As main risk factor, smoking habit was taken into account in 30 patients for which this information was available; among these, 12 patients (40%) were cigarette smokers, 5 patients (16.67%) have a history of tabagism and 13 patients (43.33%) were non-smokers (Table 1).

Urine samples were also collected from two groups of healthy donors of 4 and 6 individuals with characteristics comparable to the ccRCC patients included in the study (median age: 60.5; males: 60% and females: 40%).

### RNA extraction and microRNA expression analysis

Fresh Frozen samples were homogenized by gentle dissociator (Miltenyi Biotec) in 700 μl of Qiazol (Qiagen, Chatsworth, CA) and RNA was extracted following the manufacturer’s instruction. The concentration and purity of total RNA were assessed using a Nanodrop TM 1000 spectrophotometer (Nanodrop Technologies, Wilmington, DE, USA).

A quantity of 150 ng of total RNA was reverse transcribed in 20 μl using miScript II RT kit (Qiagen, Chatsworth) and 1 μl of cDNA dilution (1:5) was used for quantitative Real time PCR (RT-qPCR) experiments.

PCR quantification analysis of the SNORD61, SNORD68, RNU6-2 and miRNAs miR-21-5p, miR-210-3p, miR-221-3p, miR-185-5p and miR-145-5p, was performed using the miScript SYBR Green PCR kit (Qiagen, Chatsworth) with the miScript Primer Assay Hs-SNORD61 (#MS00033705), SNORD68 (#MS00033712), RNU6B-2 (#MS00033740), Hs-miR-21-5p (#MS00009079), Hs-miR-210-3p (#MS00003801), Hs-miR-221-3p (#MS00003857), Hs-miR-185-5p (#MS00003647) and Hs-miR-145-5p (#MS00003528) (Qiagen, Chatsworth, CA, USA). All reactions were performed in triplicate. Data were analyzed by quantitative Real time PCR relative to a standard curve; standard curve was performed with serial dilution of a reference cDNA obtained from RNA extracted from a tumor sample. For the analysis z-score were calculated for all expression value to standardize the data. Subsequently, z-score values of SNORD61, SNORD68, RNU6-2 were averaged and used to normalize the expression values of each miRNA. The p value was calculated by using a parametric test with paired data.

Total RNA from 200 μl of urine samples was extracted using miRNAeasy serum/plasma kit (Qiagen, Chatsworth, CA) following the manufacturer’s instructions. The concentration purity and quality of total RNA were assessed using a Nanodrop TM 1000 spectrophotometer (Nanodrop Technologies, Wilmington, DE, USA). A quantity of 25 ng of total RNA was reverse transcribed in 10 μl using miScript II RT kit (Qiagen, Chatsworth) and 1 μl of cDNA dilution (1:5) was used for quantitative Real time PCR (RT-qPCR) experiments. Quantification of miRNAs miR-21-5p, miR-210-3p, miR-221-3p was carried out by miScript Primer Assay (Qiagen, Chatsworth, CA), normalizing over the Spike-In Control (#219610) (Qiagen, Chatsworth, CA) through ΔΔCT method and using the miScript SYBR Green PCR kit (Qiagen, Chatsworth, CA). All reactions were performed in triplicate. microRNA expression was evaluated on urine samples from each of the two ccRCC cohorts and compared to that of the two groups of urine samples from healthy donors (first cohort vs. 4 donors; second cohort vs. 6 donors). Data were then collected and presented in box plots.

## SUPPLEMENTARY MATERIALS FIGURE


