# Supplementary material for: Secreted miR-210-3p as non-invasive biomarker in clear cell renal cell carcinoma
Source: Oncotarget. 2017 Jun 13;8(41):69551–8. doi: 10.18632/oncotarget.18449 (PMC5642499; doi:10.18632/oncotarget.18449)
Supplement: Supplementary file 1 [file oncotarget-08-69551-s001.pdf]

Secreted miR-210-3p as non-invasive biomarker in clear cell renal cell carcinoma

SUPPLEMENTARY MATERIAL

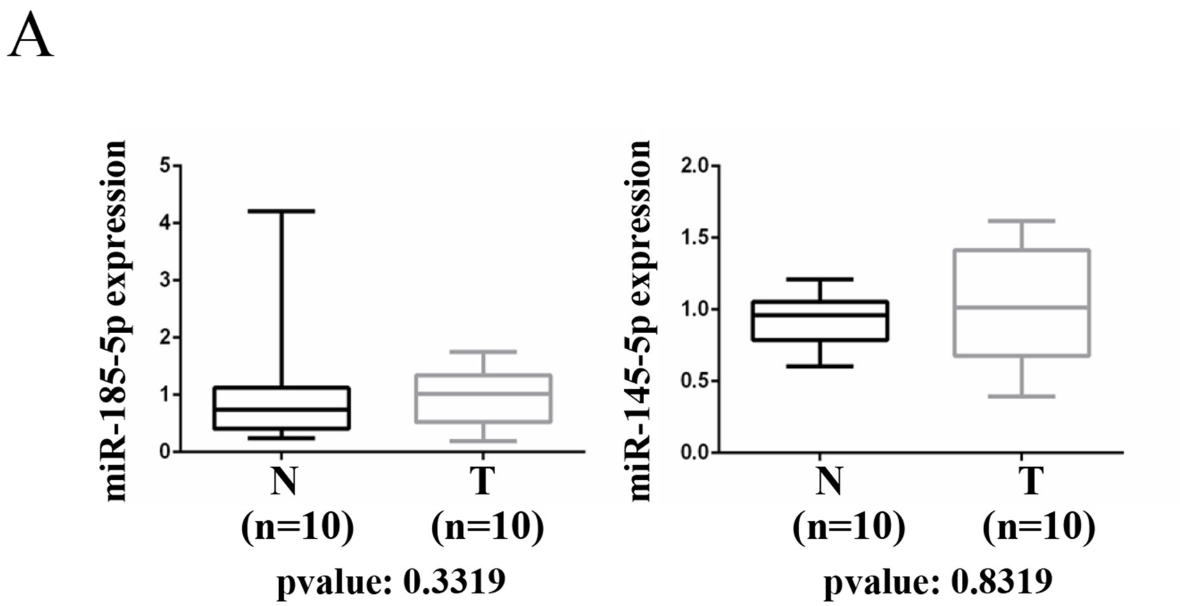

Supplementary Figure 1: Evaluation of microRNAs levels in fresh froze tissues from ccRCC patients. **(A)** Box-plot showing the modulation of miR-185-5p and miR-145-5p in a cohort of 10 matched fresh frozen tissues from ccRCC patients. A total of 10 matched ccRCC tumor (T) and adjacent normal tissue (N) samples were analyzed by RT-qPCR. The expression value of each miRNA was normalized over the average of SNORD61, SNORD68 and RNU6-2 expression through z-scores method.
